# Phylogeography and historical demography of the Pacific Sierra mackerel (*Scomberomorus sierra*) in the Eastern Pacific

**DOI:** 10.1186/1471-2156-11-34

**Published:** 2010-05-03

**Authors:** Mónica Domínguez López, Manuel Uribe Alcocer, Píndaro Díaz Jaimes

**Affiliations:** 1 Departamento de Ecología Marina. Instituto de Ciencias del Mar y Limnología, Universidad Nacional Autónoma de México, Circuito Exterior de Ciudad Universitaria/Apdo. Postal 70-305, México, D.F. 04510. México

## Abstract

**Background:**

Testing connectivity among populations of exploited marine fish is a main concern for the development of conservation strategies. Even though marine species are often considered to display low levels of population structure, barriers to dispersal found in the marine realm may restrict gene flow and cause genetic divergence of populations. The Pacific Sierra mackerel (*Scomberomorus sierra*) is a pelagic fish species distributed throughout the tropical and subtropical waters of the eastern Pacific. Seasonal spawning in different areas across the species range, as well as a limited dispersal, may result in a population genetic structure. Identification of genetically discrete units is important in the proper conservation of the fishery.

**Results:**

Samples collected from the Eastern Pacific, including the areas of main abundance of the species, presented high levels of mtDNA genetic diversity and a highly significant divergence. At least two genetically discrete groups were detected in the northern (Sinaloa) and central areas (Oaxaca and Chiapas) of the species range, exhibiting slight genetic differences with respect to the samples collected in the southern region (Peru), together with a "chaotic genetic patchiness" pattern of differentiation and no evidence of isolation by distance. Historical demographic parameters supported the occurrence of past population expansions, whereas the divergence times between populations coincided with the occurrence of glacial maxima some 220 000 years ago.

**Conclusions:**

The population genetic structure detected for the Pacific Sierra mackerel is associated with a limited dispersal between the main abundance areas that are usually linked to the spawning sites of the species. Population expansions have coincided with glacial-interglacial episodes in the Pleistocene, but they may also be related to the increase in the SST and with upwelling areas in the EEP since the early Pleistocene.

## Background

The definition of factors that determine genetic divergence in populations from marine ecosystems has become a subject of interest for evolutionary biology and conservation. The subdivision of populations in an apparently homogeneous environment has been found to be complex [1]. Evolutionary divergence processes in the marine realm are thought to have minor effects on species with long-lived pelagic eggs and larvae, and/or on those that have a significant capacity to disperse through migrations, as well as large effective population sizes. For many marine species, dispersal mediated by larval drift promotes their range expansion as well as a mixing of populations [2]. Likewise, the effects of genetic drift may be imperceptible in species with large effective population sizes [3]. Consequently, most marine species, especially pelagic species, are widely distributed with patterns that vary from disjunct to circumtropical [4]. However, larval duration and adult vagility may vary considerably among species, and determine the extent of gene flow [5]. Moreover, mesoscale physical events such as upwelling, jets, gyres, tides and fronts may affect larval dispersal and act as temporary barriers to genetic exchange through larval retention [1]. As a result of this, pelagic species may display a weak genetic structure [6].

The Pacific Sierra mackerel (*Scomberomorus sierra*) is an epipelagic fish species distributed in tropical and subtropical waters along the North, Central and South American Pacific coast. Mackerels are a diversified group consisting of 18 species distributed across the continental margins of every ocean [7]. Reported also are the sister species *S. concolor *confined to the Gulf of California in the Pacific, and *S. sierra *that is widely distributed along the eastern Pacific coast [7]. Although information on the biology of the Pacific Sierra mackerel is scarce, spawning seems to be influenced by sea surface temperatures during Spring and Summer. It takes place in geographically distinct regions that include the area off the Mexican mainland from July to September [8], in Costa Rica from late August to November, and in Colombia from November to April. Klawe [9] reported that spawning takes place from December to April in the lower latitudes of the Eastern Pacific. Asynchronous spawning in distant areas may result in the isolation of populations, and thus promote the formation of discrete genetic populations. Larvae of *Scomberomorus *species have been found to have a short larval period (8.3-9.1 days) [10]. Dispersal is moderate in the early stages of its life cycle and seems to be associated with feeding and spawning activities.

Population genetic studies on pelagic species in the eastern Pacific have reported some extent of population subdivision. Examples include yellowfin tuna [11], related mackerel species living in waters with oceanographic conditions similar to those in the eastern Pacific [12], and a congeneric species in the Gulf of Mexico, where moderate levels of genetic differences at the population level were recorded by Gold et al. [13].

The Pacific Sierra mackerel is a major fishery in Mexico with catch statistics estimated at 10,000 metric tons in 2006, and with an increasing trend in fishing effort [14]. No management strategies have been implemented for this species so far, nor has the existence of a genetic stock structure been defined. Delineation of conservation and management strategies requires information regarding the number of independent units comprising mackerel populations in the Eastern Pacific.

In order to test the hypothesis on the genetic homogeneity of Pacific mackerel, the genetic diversity of samples across the range of species was analysed using a 648 bp segment of the hypervariable mtDNA- control region. Sequence analyses consisted in applying a coalescence approach to estimate historical demographic parameters and to determine the occurrence of significant population fluctuations in the past. Pairwise migration rates and divergence times among populations were estimated through a coalescent-based approach, in order to distinguish between recent isolation and low levels of contemporary migration. Genetic differences among samples from the three different regions were recorded, in addition to the occurrence of population expansions related to the climate in the past and/or to ecological changes in the eastern equatorial Pacific.

## Results

### mtDNA region control variability and temporal stability

The analysis of a 648 bp fragment from the mitochondrial control region of Pacific mackerel *S. sierra *revealed very high levels of variation. Of 246 individuals studied, 153 variable sites consisted of 59 transitions and 10 transversions that produced 239 haplotypes, 59 of which consisted of singleton sites. Ninety-four parsimonious informative sites were identified, with nucleotide differences averaging 12.695. Overall, haplotype (*h*) and nucleotide diversity (π) for all sequences were 0.997 (± 0.0004) and 0.01959 (± 0.00857) respectively (Table 1). The high number of haplotypes was related to the presence of five doublet mutations on adjacent nucleotides at positions 141-142 (AT-TA), 218-219 (TC-CT), 247-248 (GA-AG), 318-319 (GA-AG) and 476-477 (TC-CT).

**Table 1:** mtDNA-Dloop sequence variability and mismatch parameters under demographic (τ, θ_0 _and θ_1_) and spatial expansions (τ, θ and *m*).

		Population					
		
		SIN	SIN-04	MICH	OAX	CH	PE
Genetic variability	*n*	43	26	31	50	47	49
	*nh*	42	26	28	48	47	49
	*h*	0.999	1.0	0.994	0.998	1.0	1.0
	(SD)	(0.005)	(0.011)	(0.01)	(0.004)	(0.004)	(0.004)
	π	0.0244	0.0076	0.00144	0.0183	0.0214	0.0217
	(SD)	(0.0097)	(0.0036)	(0.0048)	(0.0072)	(0.0082)	(0.0089)

Demographic	τ	1.7	5.0	10.6	9.5	13.4	11.8
	*T *(Ky)	250.8	108.3	228.3	204.9	287.2	252.8
	(CI-95%)	161.4-550.8	75.1-135.9	138.5-292.4	143.5-327.1	212.4-387.0	168.7-289.8
	θ_0_	5.1	0	0.01	3.0	1.7	0.0
	*N*_0_	55 000	0	128	32 418	18 432	0.0
	θ_1_	5.1	99 999	51.5	255.3	105.9	156.2
	*N*_1_	55000	1.07 × 10^9^	552 300	2.7 × 10^6^	1.13 × 10^6^	1.68 × 10^6^

Spatial	τ	10.4	5.0	8.9	9.2	12-0	11.5
	*T *(Ky)	223.5	108.3	191.8	198.2	258.5	246.5
	(CI-95%)	154.9-391.6	60.4-135.8	119.8-274.7	142.5-280.7	201.5-335.8	189.0-337.0
	θ	6.4	0.0007	1.5	3.3	3.0	3.1
	*N*	69 400	7.5	16 500	35 900	33 100	33 800
	M	530.3	99 999	94.2	1251.9	2006.6	99 999
	*m*	0.004	6 600	0.003	0.02	0.03	1.5

Sample size (*N*), number of haplotypes (*nh*), haplotype diversity (*h*), nucleotide diversity (π), standard deviation (SD), time since expansion (*T*) in thousand years (Ky). Ranges for *T *are given for 95% confidence intervals.

Pairwise-sample estimates of Φ_ST _revealed no differences in temporal variability among the samples collected in several years: in Oaxaca from 2004 to 2005, and in Chiapas from 2003 to 2004 (Table 2). The Sinaloa samples of 2003 and 2008 presented no differences, yet a comparison with the Sinaloa 2004 samples did indicate significant differences. The 2004 samples remained different even after the other two samples were pooled together. Thus, the Sinaloa 2004 samples were considered a different sample (SIN04), and the temporal collections with no differences were pooled into the Sinaloa (SIN), Oaxaca (OAX) and Chiapas (CH) samples for further analysis.

**Table 2:** Pairwise Φ_*ST *_estimates for population differentiation and MDIV estimates.

Comparison	TMRCA	*T*	*M*	θ	Φ_*ST*_
SIN-SIN04	13 600	104 400	1.4	69.6	0.073**
SIN-MICH	11 500	101 500	2.0	80.4	0.032*
SIN-OAX	8 800	110 000	4.8	114.0	0.045**
SIN-CH	9 300	144 000	6.1	103.7	0.015
SIN-PE	7 800	188 600	5.4	125.7	0.016*
SIN04-MICH	11 100	153 400	1.1	37.7	0.094**
SIN04-OAX	5 900	96 500	4.2	75.9	0.068**
SIN04-CH	5 600	150 400	1.7	84.0	0.081**
SIN04-PE	4 800	114 400	2.1	106.5	0.085**
MICH-OAX	11 300	41 700	9.9	78.0	0.005
MICH-CH	6 200	80 500	5.8	83.5	0.027*
MICH-PE	5 500	116 700	7.7	99.0	0.007
OAX-CH	4 900	126 700	9.9	108.0	0.035*
OAX-PE	4 700	115 700	7.5	126.3	0.019*
CH-PE	4 800	98 500	14.8	131.4	0.004

Time to the most recent common ancestor (TMRCA), time of population divergence (*T*), migration rate (M), and ancestral population size (θ). Values for *T *and TMRCA were calculated using a generational time of 2 years and mutation rates were estimated at 3.6 × 10^-8 ^substitutions/site/year [46]. Φ_*ST *_values with * were significant without Bonferroni correction for the acceptance level (α = 0.05) and values with ** remained significant after correcting for multiple testing (α = 0.5/15 = 0.003).

### Intraspecific Phylogeny

**Figure 1:**
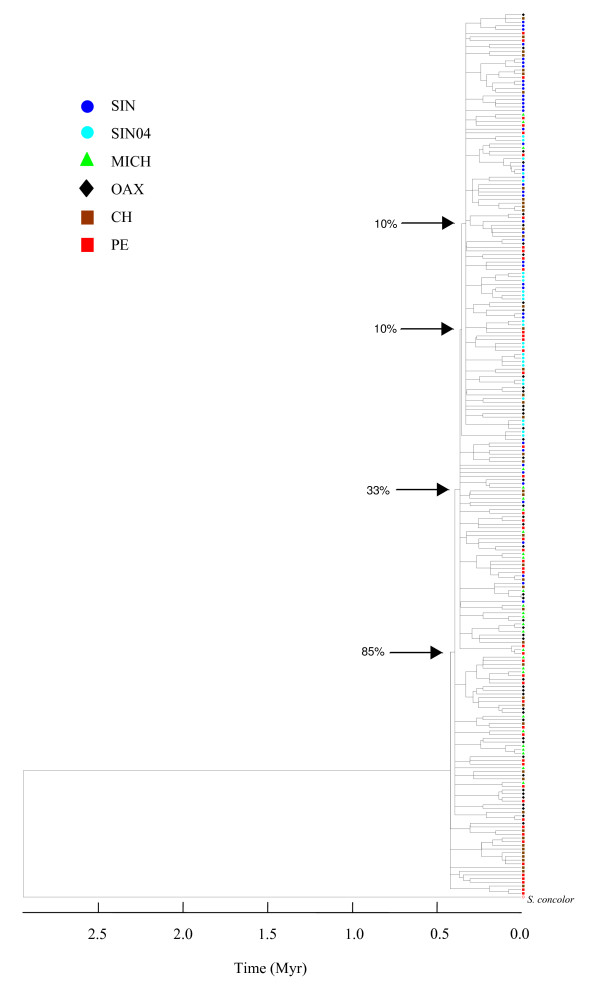
**Neighbor-joining tree of *Scomberomorus sierra *haplotypes**. Support of branches was determined by 1000 bootstrap re-sampling. The tree was linearised using the *Scomberomorus*-specific divergence rate of 2.1% per million years of Branford [34], to represent the time since the present.

Phylogeny reconstruction based on a NJ algorithm resulted in a non-obvious phylogeographic pattern with a notorious absence of clear clustering of haplotypes (Figure 1). The tree was characterised by a low support of branches and a politomic arrangement of most haplotypes that belong to the same location, but also with haplotypes mainly from the Chiapas and Peru samples (Figure 1). The external haplotype represented by *S. concolor *was clearly separated from the rest of the sequences.

### Population structure

The pairwise-sample estimates of Φ_ST _resulted in a chaotic pattern of differentiation where genetic differences were observed for some comparisons involving both, spatially close samples and samples from spatially separated localities. Moreover, sample SIN04 presented an odd pattern of differentiation with differences in relation to the other samples even after corrections for multiple testing had been made (α = 0.05/15 = 0.003) (Table 2). However, some patterns of differentiation could be identified from some of the highly significant Φ_ST _-estimates.

Estimates of pairwise sample Φ_ST _revealed highly significant genetic differences mainly for the comparisons involving the Sinaloa samples (SIN and SIN04), the Oaxaca sample, and the Peru sample (Table 2), although only the comparisons between SIN-OAX (Φ_ST _= 0.045; *P *< 0.001), SIN04-OAX (Φ_ST _= 0.068; *P *< 0.001) and SIN04-PE (Φ_ST _= 0.085; *P *< 0.001) remained significant after correcting for multiple testing (α = 0.05/15 = 0.003). Therefore, a clear divergence pattern was identified for the comparison between the locations that were most separated (SIN04 and PE), but especially between the northernmost (SIN, SIN04) and intermediate locations (OAX, CH). In contrast, no differences were detected between the southernmost sample (PE) and the intermediate samples, after correcting for multiple testing. As a result, when the AMOVA was applied to the samples grouped in the northern (SIN and MICH), central (OAX and CH) and southern (PE) regions, based on the differences found from pairwise sample Φ_ST_-estimates and proximity of sample locations, the analysis indicated there were no significant differences (Φ_CT _= -0.022; *P *= 0.95) among these groups. However, the differences among populations within groups were confirmed (Φ_SC _= 0.054; *P *< 0.001).

Additional genetic differences between some comparisons involving the SIN, OAX and PE locations, and the MICH and CH samples, were also observed. However, only the differences resulting from the comparison between the highly divergent sample SIN04, and the MICH (Φ_ST _= 0.094; *P *< 0.001) and CH (Φ_ST _= 0.081; *P *< 0.001) samples, remained significant after correcting for multiple testing.

**Figure 2:**
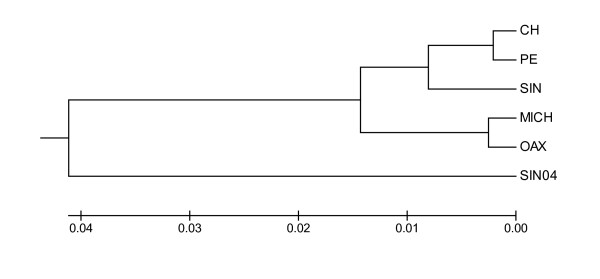
**UPGMA tree from Slatkin's linearised *F*_*ST *_genetic distances (expressed in coalescence times) between locations**.

The UPGMA tree constructed with the linearised Slatkin's *F*_*ST*_'s between samples coincided with the main differences among groups of populations, but reproduced the chaotic pattern of differentiation detected by the pairwise differences (Figure 2). The SIN04 population was clearly separated from the rest of the samples, whereas PE-CH and OAX-MICH formed two divergent geographic groups, with the SIN sample in an intermediate position between both groups (see Figure 2).

Considering the observed differences between the SIN and SIN04 samples, the isolation by distance model was verified with the Mantel test, omitting one of the Sinaloa samples and using the number of migrants by generation (*Nm*). No significant correlations were found between the distance and the *Nm *estimates (*r*^2 ^= 0.26; *P *= 0.2) when the SIN04 sample was omitted. Neither was there any correlation when the SIN sample was omitted, and the correlation was similar (*r*^2 ^= 0.18; *P *= 0.3). This pattern was clearly influenced by the slight differences observed between the SIN and the Peru samples, based on the pairwise Φ_ST _estimates.

### Historical demography

**Figure 3:**
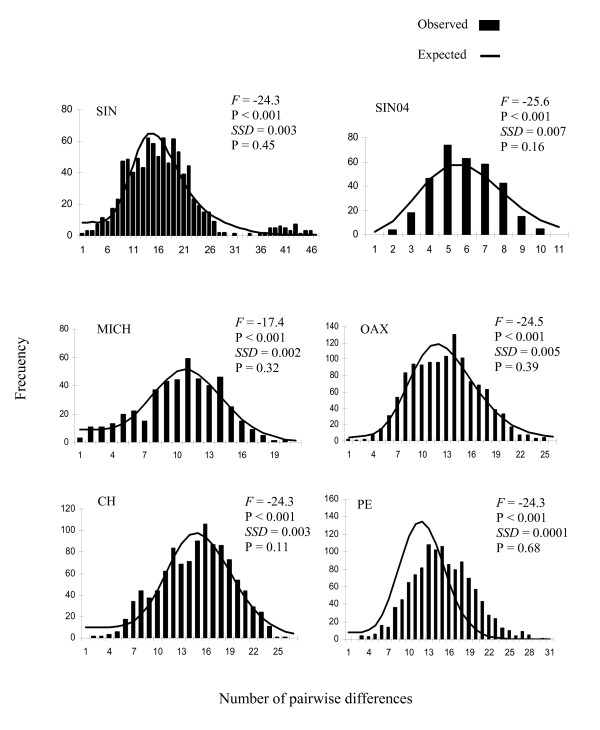
**Comparison between observed and expected pairwise mismatch distributions under an expansion model **[47]**, sum of square deviations (*SSD*) between the observed and the expected mismatch and Fu's *F *statistics, and their respective significance probabilities (*P*) **[49].

The distribution of mismatches was unimodal for all samples (Figure 3). Similarly, non significant deviations for the sum of square deviations (*SSD*) between the observed and the expected mismatch distributions were observed besides very high and significant Fu estimations (Figure 3). Considering a full demographic expansion model as the factor responsible for the observed pattern of mismatch distribution, it was estimated that the time since the expansion occurred was approximately 204 000-280 000 years ago for all the samples, with the exception of SIN04 which occurred 108 000 years ago (Table 1). The low effective population size of the females estimated for populations SIN04, MICH and PE (range 0-0.01) suggests there were drastic reductions before the expansion, followed by a sudden expansion, given the large difference between the initial number of females before (*N*_0_) and after (*N*_1_) the expansion. In contrast, the expansion was slow for the SIN, OAX and CH populations, and may have occurred from a relatively high number of females (*N*_0 _= 18 000-55 000).

Since the observed mismatch pattern may also be explained by a range expansion of the populations [15], the parameters were obtained under a spatial expansion model [16]. If spatial expansion were to be assumed as the factor responsible for the observed population molecular architecture, such an expansion could have occurred in a similar time, or even more recently, than the demographic expansion of all populations, that is to say, approximately 250,000 years ago. The range expansion of all the populations, excepting SIN04, probably originated from a significant number of females (*N *= 16 500-69 400) with a very low rate of exchange with the original population (Table 1). In contrast, the population expansion of SIN04 seems to have occurred 104 000 years ago from a reduced number of females (*N *= 7.5) with a very high migration rate (Table 1).

The extent of the difference between the estimates of the time expected since the most common ancestor (TMRCA) and the divergence time (*T*) obtained with the MDIV, may be used to distinguish short divergence times with low gene flow from long divergence times with moderate gene flow, as an explanation for population divergence [17]. Despite the fact that convergence of the likelihood function for the divergence time in all comparisons was close to zero, estimates of TMRCA were lower than *T *for all pairwise-sample comparisons (Table 2), in agreement with a model of recent isolation and reduced or no migration.

Larger differences were observed between estimates of *T *in relation to TMRCA for comparisons involving samples from the northern (Sinaloa), central (OAX) and southern (Peru) groups of samples (Table 2). However, small differences were observed between *T *and TMRCA (41 700 and 11 300 years respectively) in the MICH - OAX comparison, suggesting a re-establishment of gene flow after isolation. In general, populations from the northern group (SIN and SIN04, except MICH) and the southernmost sample (PE) diverged early from the central populations (OAX and CH).

The coincidence in the divergence time and the time since the occurrence of the population expansion must be highlighted. The separation of populations, except for SIN04 which expanded more recently, took place after the demographic or spatial expansion event, suggesting a possible relation between divergence and the drastic reduction caused by glacial events that occurred during the Pleistocene.

One may also obtain estimates of the ancestral population size (θ) and migration (M) for all pairwise-sample comparisons from the MDIV. Estimates of θ were high (37.7-131.4), indicating that populations originated from relatively large ancestral population sizes. For the various θ estimates between population pairs, those involving the Peru sample presented the largest values, indicating that this was the ancestral population (Table 2).

Migration estimates between populations from the northern group (SIN, SIN04 and MICH) were low in relation to the central samples (1.7-9.9) and the southern sample (2.1-7.7), and were consistent with a model of divergence that resulted from restrained gene flow. Migration estimates between the central and southern populations were higher (7.5-14.8), and this, together with the similarity in divergence times and the modest levels of differences among both groups observed, suggests there was a secondary contact between populations after divergence.

## Discussion

### Genetic variability and temporal stability

The analyses of a 648 bp fragment of mtDNA-Dloop in the present study revealed high levels of genetic diversity in the analyzed populations. The highly significant genetic diversity in terms of haplotypes for *S. sierra *could be explained by the very high estimates of ancestral effective population size in conjunction with recent expansion events.

The differences observed among some Sinaloa samples of different years, and the lack of temporal sampling in Peru, made it impossible to be fully conclusive about the temporal genetic stability of the Pacific mackerel population, regardless of the homogeneity between the temporal collections of the Oaxaca and Chiapas populations, as well as for the 2003 and 2008 Sinaloa samples. The 2004 Sinaloa samples exhibited marked differences with respect to the 2003 and the 2008 Sinaloa samples, and to the rest of the samples. They presented notoriously different molecular diversity indices (Table 1) with a low level of nucleotide diversity (0.76%; average 2%), in addition to a diversity in the mean number of segregating sites (24; mean 70.1) and the mean number of pairwise differences (4.9; average 13.3). Such diversity indices are evidently related to the differences observed. This odd population structure where temporal genetic differences and/or small scale spatial differences are observed, is recurrent for fish in a wide variety of ecosystems, although it has been particularly identified for marine species and attributed to non-random sampling in a phenomenon called "chaotic genetic patchiness" [18]. In the marine environment, offspring of species with large spawning aggregations may present a differential survival success (sweepstakes-chance matching hypothesis). Hence, for a given year, most of the recruited young may be generated from a reduced number of adults, usually displaying low levels of genetic variability [19]. Moreover, the size selective sampling methods used by fisheries tend to over-represent some cohorts. Hence, the genetic data collected from this sort of samples will depend on a subset of parents in a generation (e.g., a particular cohort), biasing the estimation of population differentiation upwards. The pattern of molecular diversity displayed for the SIN04 sample was consistent with this evidence. Furthermore, differences in age composition between SIN04 and the other samples was observed; whereas age 2 was notoriously over-represented (74%) in relation to age 3 (22%) in the SIN04 sample, the two ages were equally represented in the SIN sample (50% for age 2 and 3 respectively) as well as in age composition for the MICH (53% for age 3, 44% for age 4 and 3% for age 5) and the OAX (47% for age 2 and 53% for age 3) samples. Unfortunately, data on the number of cohorts that were represented in each sample was not available for all the samples.

Despite the evidence recorded for the age differences, the evidence observed for the "chaotic genetic patchiness" that resulted from the non-random sampling of cohorts of Pacific Sierra mackerel, require further examination and should be considered for future studies.

### Population genetic structure

Despite the "chaotic genetic patchiness observed", the pairwise-sample divergence estimates indicated the existence of clear differences between the samples from the northern (SIN), central (OAX) and southern (PE) areas of the eastern Pacific, and especially between two of the areas of greatest abundance (Sinaloa and Oaxaca) that are also regarded as spawning areas in the Mexican Pacific [8,9]. Regardless of the fact that the AMOVA failed to determine differences among the groups, at least two genetically differentiated units across the species range distribution can be considered based on the pairwise haplotype divergence estimates: a northern population localised at the mouth of the Gulf of Baja California (Sinaloa), a second population in the Gulf of Tehuantepec (Oaxaca), and a possible third population off Peru as no consistent differences were found between the OAX and PE samples. Moreover, the existence of additional populations within the range distribution of the species cannot be discarded, as collections did not include sampling in the northernmost area of the Pacific Sierra mackerel distribution (off Baja California), or sampling of populations off Central America. The lack of significance detected with the AMOVA resulted from the differences among populations within groups, as those observed between Sinaloa-Michoacán and Chiapas-Oaxaca.

Considering that Pacific mackerel exhibit migratory behaviour, the observed population structure could be related to specific spatial or temporal spawning regimes; spawning off Mexico takes place in July, August and September, and in lower latitudes it may occur from December through April [9].

Adult mackerel have a limited capacity to migrate as a result of their body density, short pectoral fins and body length, in comparison with the large pelagic scombrids [20]. Besides, their spawning has been associated with areas of high productivity where increased larval growth and survival has been recorded [21]. Thus, we suggest that the divergence that was observed may result from a fidelity to spawning sites or a limited dispersal, rather than to adaptations to particular oceanographic regimes.

Several important upwelling areas in the Eastern Pacific include regions off Baja California, Sinaloa, Oaxaca, Panama and Peru. Given that larval survival of *Scomberomorus *species is strongly dependent on the availability of food [10], upwelling areas may represent major spawning sites for Pacific Sierra mackerel populations. As high productivity significantly increases larval growth in scombrid fish, a reduction in the duration of the larval stage, and hence of larval drift, may result in a limited dispersal from the spawning site. Consequently, a fidelity to upwelling areas and/or a limited dispersal may constitute important factors that promote isolation in Pacific mackerel populations.

The northern and central populations of our study showed well differentiated units. However, there were differences recorded between the central and the Peru populations that were not consistent after the Bonferroni correction. These populations ought to be re-evaluated since they seem to reflect independent evolutionary histories that should not be underestimated. Similarly, as gene flow estimates from MDIV were substantially lower between the northern and central populations than between the central and southern populations (Peru), gene flow could be reestablished as a result of a secondary contact favored by an increase in the number of high productivity areas in the eastern equatorial Pacific, and by the intense southern currents during the last glacial maxima [22] that might have promoted the displacement of southern populations to equatorial areas.

### Past climate changes and size fluctuations in populations

The mismatch analyses of the molecular variation were consistent in supporting the occurrence of expansions of Pacific Sierra mackerel populations after drastic reductions since unimodal mismatch distributions, as well as non significant deviations for the sum of square deviations (*SSD*) were observed. The estimations of the divergence time and the time of occurrence of population fluctuations are controversial since these are based on the accuracy of the molecular clock used. Generalized mutational rates estimated for other similar species were used, in the absence of available data for calibration. Thus, these estimates may be inaccurate when determining the exact time of occurrence of events, and the divergence time and/or time since population expansions occurred should be considered rough approximations. However, the pattern for the occurrence of these events will remain, even though departures from the mutational rate that was used to estimate divergence times might result in either deeper or shallower estimations.

Evidence of the occurrence of both demographic and/or spatial expansion events is related to glacial episodes during the last 0.5 million of years (my) in the equatorial Pacific [23]. The time of population expansions is registered as having occurred during the Yarmouth interglacial [24] some ~300-265 ky ago after the Kansas glacial stage [25]. The sea surface temperature (SST) in the eastern equatorial Pacific was about 3° to 5°C colder during the glacial cycles, and about 1°C warmer during the interglacial stage, than the conditions that have prevailed during the last 500 ky [26]. Studies on the species composition of planktonic foraminiferal and radiolarian assemblages have reconstructed the conditions of the surface waters in the eastern equatorial Pacific (EEP) during glacial-interglacial periods in the late Pliocene and mid-Pleistocene [27-29]. These studies have documented substantial fluctuations in the levels of primary productivity related to the thermocline depth in the eastern equatorial Pacific during glacial events in the Pleistocene, besides a trend in increased SST and upwelling areas in the EEP. Since Pacific mackerel rely on the presence of high productivity areas and their distribution depends on the sea temperature, reductions in SST during glacial events, combined with fluctuations in primary productivity, might have reduced their populations drastically, whereas the warmer and more productive waters of the interglacial episodes might have promoted population expansions. Therefore, the occurrence of glacial-interglacial cycles during the Pleistocene may have caused cycles of expansion-contraction of populations, whether demographic or spatial, thereby notably influencing the molecular architecture of populations. The glacial-interglacial events probably caused a similar number of reduction-expansion cycles. Both demographic and/or spatial expansion events are not mutually exclusive; they probably occurred on numerous occasions, but the mismatch results allow only the most recent event to be recorded. Because of the decrease in SST in both hemispheres, reductions in the population ranges during glacial events of the late Pleistocene have been documented for many temperate species in the Eastern Pacific [30,31].

However, because the time for the occurrence of expansions is a rough estimation, the coincidence of an expansion and an interglacial event is also approximate. Alternative explanations for population expansions of species in the eastern equatorial Pacific could be suggested. Evidence based on paleoceanographic studies has documented a trend for an increase in the SST and upwelling areas in the EEP since the early Pleistocene [28,32]. Thus, Pacific Sierra mackerel populations may have found favourable conditions for population expansions since long ago. Similarly, a heightened intensity in the Peru Current during the last glacial maxima has also been documented [23], suggesting greater possibilities of expansion to new areas.

The mismatch analyses were also consistent with the continent island model of spatial expansion, which coincides with the development of favourable conditions in the EEP. Small peripheral isolates of populations might possibly have broken away from the ancestral population to establish divergent populations in new areas. Ancestral populations are characterised by their larger sizes [33]. The pairwise sample estimates of ancestral population sizes obtained in our study were large for the comparisons involving the Oaxaca and Peru samples. Therefore, the populations located in the southern range of the species distribution (Central or South America) can be considered ancestral. The origin of *S. sierra *has been established in the *Scomberomorus regalis *group that formed in the eastern Atlantic and dispersed into the eastern Pacific through the Trans-isthmian channel before the closure of the Panamian isthmus [34] (eastern Pacific/eastern Atlantic track) [35]. The ancestral *S. sierra *population could then be located in Central America, and the northern and southern populations probably derived from this ancestral population as the result of a dispersal process following the colonisation of new areas. Our evidence reveals that population expansions associated with historical events, whether climatic (glacial-interglacial cycles) and/or ecological during interglacial periods or glaciations, have had a prominent role in the evolutionary history and in the determination of the present molecular architecture of the Pacific Sierra mackerel populations.

### Conservation

The evidence supported by the mtDNA makes it possible to recognise at least two genetically discrete population units in the distribution area of *S. sierra*. Nevertheless, due to the generalised perception of the mtDNA as a single locus having an independent evolutionary history, one must verify the imprint that climatic events have left on nuclear DNA in order to confirm the evidence found. Given the fact that no management strategies have been implemented for this species, the need to establish independent exploitation regimes for each of the population units identified should be evaluated. The effective female population size of the entire Pacific mackerel population was estimated at 1.9 × 10^6 ^females. Furthermore, the census population size may be extrapolated from catch statistics: considering a mean catch of 10 000 metric tons [14] and an estimated average weight per individual of 450 g, the calculated number of harvested fish can be fixed at 22.2 × 10^6^. Actual levels of exploitation are ten times the effective population size of females, amounts which might have contributed to setting the population at the overexploitation status as reported by the biological indicators for the Pacific sierra fishery in Mexico [8]. Thus, there is an imperative need for effective management strategies in the conservation of Pacific Sierra mackerel.

## Conclusions

The analyses of a fragment of mtDNA-Dloop for *Scomberomorus sierra *revealed the existence of at least two genetically differentiated units across the species range distribution in the eastern Pacific, which corresponds to main spawning areas for the Pacific Sierra mackerel. These areas should be subject to independent exploitation regimes in order to preserve the genetic integrity of the populations. The observed population structure may be the result of fidelity to spawning sites and/or of a limited dispersal, even though evidence was also found of contemporary gene flow between the central and southern areas. Mismatch analyses exhibited substantial fluctuations in the population size in the past in response to climatic changes caused by glaciations or to significant changes in oceanographic conditions in the eastern equatorial Pacific during the Pleistocene. These events have left their imprint on the molecular architecture of Pacific mackerel *S. sierra, *as well as on a variety of species in similar habitats.

## Methods

### Sample collection, DNA extraction and sequencing

**Figure 4:**
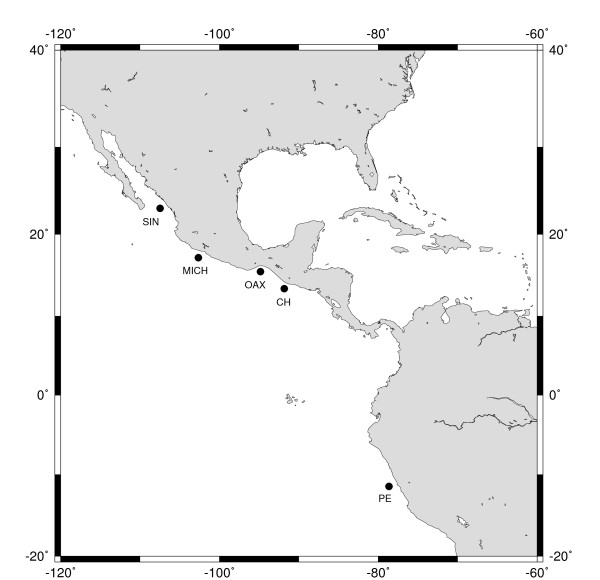
**Pacific Sierra mackerel sampling sites**. Sinaloa (SIN), Michoacán (MICH), Oaxaca (OAX), Chiapas (CH), Peru (PE). The map was designed by using the Online Map Creation facility http://www.aquarius.geomar.de/.

Tissue samples of Pacific mackerel *S. sierra *were collected at four locations over five consecutive years from small (artisanal) fishing boats operating on a local scale in the eastern Pacific (Figure 4). The sampling locations comprise almost the entire species range: Sinaloa in 2003 (SIN03; n = 23), 2004 (SIN04; n = 26) and 2008 (SIN08; n = 20); Michoacán in 2002 (MICH02; n = 31); Oaxaca in 2003 (OX03; n = 25) and 2005 (OX05; n = 25); Chiapas in 2003 (CH03; n = 21) and 2004 (CH04; n = 26) and Peru in 2005 (PE05; n = 49). The fork length of individuals from Sinaloa, Michoacán and Oaxaca, were obtained and compared with the size-distribution modes of Aguirre-Villaseñor [8] for *S. sierra *to determine the age composition of samples. Total genomic DNA was isolated using the proteinase K - lysis-buffer extraction [36] and resuspended in 50 - 100 μL of TE buffer.

The complete sequence of the mitochondrial region control of mackerel was amplified (900 bp) by using the primers Pro5-M13 (5'-TAATCCTGCCGCAATTATCC-3') and Teleost (5'-AGGCCTTCCAGGTTAGGTGT-3') reported in Carlsson et al. [37]. Sequences were aligned and used in designing internal primers so as to amplify a 750 bp segment with the polymerase chain reaction (PCR) in a total of 246 fish [GenBank accession numbers: GQ329882-GQ330120]. Reactions for sequencing were made in total volumes of 50 μL containing 50-100 ng DNA, in amplification buffer, 10 mM TRIS-HCl (pH 8.4), 50 mM KCl, 1.5 mM MgCl_2_, 0.2 mM of each dNTP, 0.1 mM of each primer and 2.5 units of platinum *Taq *DNA polymerase (Invitrogen, Cat. 10966-030). PCR amplifications consisted of 35 cycles of 1 min at 95°C for denaturalisation, 1 min at 59°C for annealing, and a final extension at 65°C for 3 min. PCR products were purified with either a QIAquick purification kit (QIAgen No. Cat. 28104) or the ChargeSwitch PCR Clean-Up kit (Invitrogen, Cat. CS12000). Purified products were sequenced in the forward direction on an ABI 3730xl automated sequencer applying the dye-termination method (Applied Biosystems). Pairwise alignment was made between each individual sequence and the most frequent haplotypes for every location in order to solve ambiguities and/or confirm nucleotide assignments.

### Intraspecific Phylogeny

Multiple alignment was performed with ClustalX ver. 1.8 [38] as well as by optimising the gap penalties in order to minimise artificial homologies between haplotypes (homoplasy) during the alignment. The hierarchical likelihood ratio method implemented in MODELTEST 3.06 [39] was applied to define the most appropriate substitution model of sequence evolution for the segment of the mtDNA-Dloop analysed. Haplotype (h) and nucleotide (π) diversities were estimated using Arlequin 3.0 [40] and with the application of the Tamura-Nei substitution model [41] as selected by the MODELTEST. A Neighbour-joining tree (NJ) based on Tamura-Nei distances was constructed using the Mega version 4.0 software [42] in order to test the evolutionary relationships among the mtDNA haplotypes. Trees were rooted with *S. concolor *sequences, and a bootstrap resampling [43] based on 1000 replicates was used to determine the statistical support for the nodes. In order to define relationships among the populations, linearised Slatkin's *F*_*ST*_'s expressed in terms of the coalescence times [44] were estimated in accordance with Arlequin. This considers a demographic model where two haploid populations of size *N *diverged τ generations ago from a population of identical size without exchanging any migrants. A UPGMA tree was generated using the coalescence times among locations using Mega 4.0.

### Population structure and historical demography

The Arlequin software package was used to obtain the unbiased estimator of Wrights *F*-statistics Φ_ST _(an mtDNA analogue for *F*_ST_), for hierarchical *F*_ST _[45], and pairwise sample Φ_ST _analyses (20100 permutations) on mtDNA sequence data. The Φ_ST _analyses were performed using a matrix of Tamura-Nei distances, with a gamma shape parameter of 0.4.

The demographic parameters τ, θ_0_, and θ_1 _were obtained from nucleotide mismatch distributions with the Arlequin software in order to examine the impact of demographic fluctuations related to past glaciations on the molecular architecture of Pacific mackerel populations. Considering the significant Φ_ST _estimates within populations, samples were analysed separately. As regards demographic expansion, the parameter Tau (τ) is an estimator of the mutational time since the occurrence of population expansion, that can be used to obtain estimates of the actual time (*T*): this is true as population expansion by *T *= 2μ/τ, where μ is the mutation rate estimated at 3.6%/Ma, for the mtDNA control region of "germinate species" of snook of the genus *Centropomus *[46]. Estimates of effective female population size for an initial population *N*_0_, assumed to grow to size *N*_1 _under Roger's sudden expansion model [47], were obtained by substituting θ_0 _= 2μ*N *and θ_1 _= 2μ*N *where μ is the mutational rate considering a generation time of 2 years for Pacific mackerel [48]. Mismatch analyses may also provide information regarding the range expansion of populations given that a unimodal distribution of pairwise nucleotide differences among sequences may leave a similar molecular architecture for both events [15,16]. Therefore, we conducted an analysis to estimate the three spatial expansion parameters τ, θ = θ_0 _= θ_1_, assuming that *N *= *N*_0 _and *M *= 2*Nm *where *m *is the rate at which the sampled deme would exchange migrants with a single population of infinite size; we used the same methods to estimate the demographic expansion parameters as implemented in Arlequin. The estimate of the effective number of females needed to explain the actual mismatch distribution under the spatial expansion model is given by N = θ/2μ. Finally, Arlequin was also used to obtain the sum of square deviations (*SSD*) between the observed and the expected mismatch as a statistical test for the fit to a unimodal mismatch distribution and to calculate estimates of Fu's neutrality test (*F*_*S*_) and explore whether mutations should be considered neutral or influenced by selection where significantly negative values are also indicative of population expansion [49].

The Isolation by Distance model (IBD) [50] was tested considering the absolute number of migrants exchanged between two populations estimated as M = 1- F_ST_/2F_ST _(assuming that M = 2 Nm for haploid data). This was done to evaluate the correlation of the distance between localities measured as a geographic separation in nautical miles (nm), and the M values obtained by means of the Mantel test [51] as implemented in Arlequin.

The migration rate and the divergence time from pairwise-sample comparisons were obtained with the Markov chain Monte Carlo approach as implemented in the MDIV software to test the "isolation with migration" model [18], in order to distinguish between a recent isolation and low levels of contemporary migration. The model parameters were θ (θ = 2*N*_*ef*μ_) where *N*_*ef *_is the effective population size and μ is the mutation rate, *M *(M = 2*N*_*ef *_*m*) where *m *is the migration rate, and *T *(*T *= *t*/*N*_*ef*_) where *t *is the divergence time. These parameters were obtained using a finite sites model (HKY). The length of the Markov chains consisted of 5,000,000 cycles with a burn-in time of 500,000. Likelihood values for θ, *M *and *T *were those displaying the highest posterior probabilities.

## Authors' contributions

MDL participated in sample collection, carried out the laboratory work, designed primers, worked in the sequence optimisation and drafted the manuscript. MUA and PDJ designed the study, supervised the genetic studies, analysed data and improved the manuscript. All authors read and approved the final manuscript.
